# Gastrodin Protects Cardiomyocytes from Anoxia/Reoxygenation Injury by 14-3-3*η*

**DOI:** 10.1155/2018/3685391

**Published:** 2018-07-25

**Authors:** Meifang Zhu, Wei Deng, Suhong Di, Mingming Qin, Dan Liu, Bo Yi

**Affiliations:** ^1^Jiangxi Provincial Key Laboratory of Basic Pharmacology, School of Pharmaceutical Science, Nanchang University, Nanchang 330006, China; ^2^Key Laboratory of Women's Reproductive Health of Jiangxi, Jiangxi Maternal and Child Health Hospital, Nanchang 330006, China; ^3^Second Abdominal Surgery Department, Jiangxi Province Tumor Hospital, Nanchang 330029, China

## Abstract

Gastrodin (GAS) is the major component isolated from the rhizome of the Chinese traditional medicinal herb “Tianma.” Many clinical studies have found that GAS protects cardiomyocytes in cardiovascular diseases, although the effects and underlying mechanisms on cardiovascular anoxia/reoxygenation (A/R) injury remain unknown. This study is aimed at exploring the effect of gastrodin on cardiomyocytes in A/R injury. Our results suggested that the protective effect of GAS on cardiomyocytes is associated with upregulated 14-3-3*η* levels. Pretreatment with GAS could increase the cell viability and decrease the activities of creatine phosphokinase (CPK) and lactate dehydrogenase (LDH). GAS could also reduce reactive oxygen species (ROS) production, inhibit mitochondrial permeability transition pore (mPTP) opening, alter the maintenance of the mitochondrial membrane potential (∆*Ψ*m), decrease the activation of caspase-3, and finally restrain cell apoptosis. Downregulating 14-3-3*η* levels by transfection with siRNA14-3-3*η* clearly attenuated the protective effect of GAS on cardiomyocytes in A/R injury.

## 1. Introduction

Myocardial ischemia/reperfusion (I/R) is an important complication of reperfusion therapy for myocardial infarction (MI). Ischemia/reperfusion (I/R) injury is a major cause of death and disability worldwide [1]. Gastrodin (4-hydroxybenzylalcohol4-O-beta-D-glucopyranoside) is a water-soluble natural compound extracted from the root of *Gastrodia elata* Blume (Orchidaceae), an ancient Chinese herbal medicine with a very long history of clinical application. The active compounds of GAS have wide-reaching biological activities, including sedative, anticonvulsive, antiepileptic, hypnotic, antidepressant, antianxiety, antipsychotic, neuroprotective, antivertigo, anti-inflammatory, circulatory system-modulating, memory-improving, analgesic, antioxidative and antiaging, antivirus, and antitumor effects [2]. However, the detailed molecular mechanisms underlying the cardioprotective effects of GAS on A/R-induced injury have not been reported previously.

The 14-3-3 protein is an acidified and highly conserved protein family found in all eukaryotic organisms, particularly in the mammalian brain. This protein is made up of seven isoforms and marked with seven separate genes, each replaced by a Greek letter (*β*, *γ*, *ε*, *ζ*, *η*, *τ*, and *σ*) [3]. The 14-3-3 proteins are highly conserved molecular chaperones connected with the regulation of important cellular functions, which include the stress response, metabolism, signal transduction, protein trafficking, cell-cycle control, transcription, apoptosis, and neurotransmission [4]. Previous studies have shown that the 14-3-3*η* protein acts as an endogenous cardioprotector and limits the development of diabetic cardiomyopathy by limiting myocardial apoptosis, hypertrophy, fibrosis, and endothelial dysfunction via the inhibition of Ask1 activation after the induction of experimental diabetes [5–8]. Therefore, the objective of this study was to investigate if the protective effects of GAS on cardiomyocytes against A/R-induced injury might be associated with 14-3-3*η*.

## 2. Materials and Methods

### 2.1. Reagents

GAS (purity: >97.6%) was purchased from the National Institute for the Control of Pharmaceutical and Biological Products (Beijing, China). The anti-14-3-3*η* antibody was from Abcam, the anti-*β*-actin antibody was from Nanjing Jiancheng Bioengineering Institute (Nanjing, Jiangsu, China), and the siRNA14-3-3*η* adenovirus was from Shanghai GeneChem Co. The LDH and CPK kits were purchased from Nanjing Jiancheng Bioengineering Institute (Nanjing, Jiangsu, China).

### 2.2. Cell Culture

The H9c2 cell line, which was obtained from the American Tissue Culture Collection (Manassas, VA), was an adherent rat myocardial cell line with myoblast morphology [9]. The cells were cultured as a monolayer in Dulbecco's modified Eagle's medium (DMEM) with 10% (vol/vol) fetal bovine serum. The medium was made up of glucose (4.5 g/L), with 17 mM of NaHCO3, 100 U/mL of penicillin, and 100 *μ*g/mL of streptomycin. Cells were incubated at 37°C in 95% humidity and 5% CO_2_.

### 2.3. Experimental Groups and Treatments

The H9c2 cells were randomly divided into different experimental groups as follows:

1. Control group: H9c2 cells were incubated under the previously described conditions.
2. A/R group: H9c2 cells were cultured in a fresh anoxia medium (1.8 mM CaCl_2_, 20 mM HEPES, 10.0 mM KCl, 1.2 mM MgSO_4_, 98.5 mM NaCl, 6.0 mM NaHCO_3_, 0.9 mM NaH_2_PO_4_, 40 mM sodium lactate, pH 6.8, 37°C), and hypoxia was induced by placing the cells in a hypoxic chamber where oxygen levels could be monitored (Biospherix ProOx C21, Lacona, NY, USA) for 3 h. After 3 h of anoxia, the cells were replaced with reoxygenation medium (1.8 mM CaCl_2_, 5.5 mM glucose, 20 mM HEPES, 5.0 mM KCl, 1.2 mM MgSO_4_, 129.5 mM NaCl, 20 mM NaHCO_3_, 0.9 mM NaH_2_PO_4_, pH 7.4, 37°C) and incubated in an atmosphere of 95% O_2_ and 5% CO_2_ for 2 h.
3. Concentration-effect GAS + A/R group: H9c2 cells were pretreated with GAS (5,10, 20, 40, 80, 160, and 320 mg/L) 24 h before the induction of A/R.
4. GAS + A/R group: H9c2 cells were pretreated with 20 mg/L GAS 24 h before the induction of A/R.
5. NC + GAS + A/R group: H9c2 cells were transfected with NC for 36 h and pretreated with 20 mg/L GAS 24 h before the induction of A/R.
6. siRNA14-3-3*η* + GAS + A/R group: H9c2 cells were transfected with siRNA14-3-3*η* for 36 h and pretreated with 20 mg/L GAS 24 h before the induction of A/R.

### 2.4. MTS Assay

Cell viability was evaluated by the 3-(4,5-dimethylthiazol-2-yl)-5-(3-carboxymethoxyphenyl)-2-(4-sulfophenyl)-2H-tetrazolium (MTS) assay (Cell Titer 96 Aqueous One Solution, Promega, Milan, Italy). H9c2 cells were cultured in 96-well plates at a density of 1 × 10^4^ cells per well. The MTS reagent (20 *μ*L) was added to the cells in each well followed by incubation for 2 h in a 37°C and 5% CO_2_-humidified incubator following the manufacturer's instructions. After 2 h, the absorbance was measured at a wavelength of 490 nm using a microplate reader (Bio-Rad Pleasanton). The results were expressed as % changes with respect to controls considered equal to 1.

### 2.5. LDH and CPK Activity Assay

LDH and CPK can be released from cells due to cell necrosis; therefore, the activity of LDH and CPK is used to evaluate the presence of cell death [10]. The supernatant was used to measure the LDH at 440 nm and the CPK at 660 nm according to the manufacturer's instructions [11, 12], and the absorbance was detected using an automatic microplate reader (PerkinElmer, USA).

### 2.6. Annexin-V/PI Assay

It has been published that apoptosis should be assessed by a flow cytometer with an Annexin V-FITC Apoptosis Detection kit (KeyGen Biotech, Jiangsu, China) [13]. After A/R treatment, H9c2 cells were collected and washed twice with phosphate-buffered saline (PBS) and then resuspended in binding buffer (500 *μ*L). Annexin V-fluorescein isothiocyanate (V-FITC, 5 *μ*L) and propidium iodide (PI, 5 *μ*L) were added to the cells (a concentration of 1 × 10^6^ cells/mL) before incubating for 15 minutes in the dark at room temperature. Then, the apoptotic cells were detected within 1 hour using a flow cytometer (BD Biosciences, San Jose, CA, USA).

### 2.7. TUNEL Assay and Hematoxylin Eosin (HE) Staining

TUNEL staining is used for detecting apoptosis. H9c2 cardiomyocytes were cultured in 24-well plates. After the treatment, the cardiomyocyte apoptosis was determined by a TUNEL kit (Promega, Madison, WI, USA), according to the manufacturer's instructions. Cells counterstained with hematoxylin were examined under a light microscope. Apoptotic cells were identified by positive TUNEL staining. Apoptotic cells are stained brown. Images were visualized on an Olympus microscope using a 10x objective.

### 2.8. Measurement of Intracellular Reactive Oxygen Species (ROS)

The formation of reactive oxygen species (Applygen, Beijing, China) was tested by flow cytometry using DCFDA (2′,7′-dichlorofluorescin diacetate) as described previously [14]. After treatment, cells were collected and washed twice with PBS, then incubated with DCFHDA (10 *μ*m/L) at 37°C in the dark for 20 min, and finally measured with a flow cytometer.

### 2.9. Western Blot Analysis

Total proteins were separately extracted. The protein content was determined using the BCA protein assay kit (Beyotime Biotechnology, Shanghai, China) [15]. Total protein lysates were separated by 10% sodium dodecyl sulfate polyacrylamide gel electrophoresis (SDS-PAGE) and transferred to polyvinylidene fluoride (PVDF) membranes and blocked with 5% nonfat milk in TBST buffer for 2 h. The membranes were then incubated with primary antibodies (1 : 500) against *β*-actin and 14-3-3*η* at 4°C overnight, washed three times with TBST, and incubated with the horseradish peroxidase-conjugated secondary antibody (1 : 2000) at room temperature for 2 h. The blots were visualized using chemiluminescence, and the final data showed the ratio between protein intensities in treated cells and the control group.

### 2.10. Determination of ∆*Ψ*m

The method for measuring JC-1 (KeyGen Biotech, Jiangsu, China) was used to assess the mitochondrial membrane potential. After treatment, the cells were collected, washed twice with PBS, and then harvested and incubated with 200 *μ*mol/L JC-1 for 30 min in the dark at 37°C. Before being tested by flow cytometry in the FL1 and FL2 channels, the samples were resuspended in 500 *μ*L of incubation buffer. The results are expressed as a relative red/green fluorescence ratio.

### 2.11. Caspase-3 Activity Assay

The activity of caspase-3 was measured following the protocol provided by the manufacturer of the caspase-3 activity assay kit (KeyGen Biotech, Jiangsu, China). The protein concentration was measured by the Bradford method, with each group with 150 *μ*g of protein absorbed in cell lysate. After adding a mixture of 50 *μ*L of 2X reaction buffer, 5 *μ*L caspase-3 substrate was added to a 96-well microtiter plate and incubated at 37°C for 4 hours. We then detected the absorbance at 405 nm.

### 2.12. Opening of the mPTP

We previously published how to isolate permeability transit pores (mPTP) and to detect mitochondria [16]. The mitochondria of the cardiomyocytes are isolated with a mitochondrial/cytosolic fractionation kit (Qiagen, Hilden, Germany) and then resuspended in the swelling buffer (120 mM KCl, 5 mM KH_2_PO_4_, 20 mM MOPS, and 10 mM Tris-HCl (pH 7.4)) and added to 200 *μ*mol/L CaCl2. The absorbance is measured at a wavelength of 520 nm, and the change rate in the mitochondrial optical density is indicated by the degree of mPTP opening.

### 2.13. Statistical Analysis

All data are expressed as the mean ± SEM and were evaluated by analysis of variance (ANOVA). *P* ≤ 0.05 is considered to indicate a statistically significant difference.

## 3. Results

### 3.1. GAS Increases the Viability of Cardiomyocytes That Have Suffered from A/R

**Figure 1:**
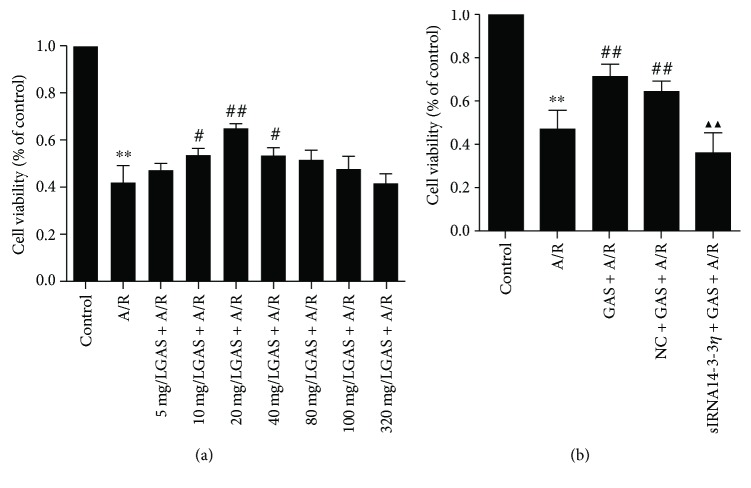
Effects of gastrodin (GAS) on cell viability of H9c2 cells after A/R. 3-(4,5-dimethylthiazol-2-yl)-5-(3-carboxymethoxyphenyl)-2-(4-sulfophenyl)-2H-tetrazolium (MTS) assay results showing that (a) the A/R group showed decreased cell viability of H9c2 cells (^∗∗^*P* < 0.01 versus control group). Pretreatment with different concentrations of GAS and the 20 mg/L GAS + A/R group exhibited the highest cell viability (^#^*P* < 0.05 and ^##^*P* < 0.01 versus A/R group). (b) The A/R treatment could decrease the cell viability of cardiomyocytes (^∗∗^*P* < 0.01 versus control group) and both the GAS + A/R group and the NC + GAS + A/R group had increased cell viability (^##^*P* < 0.01 versus A/R group), while siRNA14-3-3*η* abolished the effects of GAS on cell viability (^▲▲^*P* < 0.01 versus GAS + A/R group). Data are expressed as the mean ± SEM (*n* = 3).

We examined the viability of H9c2 cells using the MTS assay. As shown in Figure 1(a), anoxia reoxygenation injury decreased the cell survival rate significantly. Pretreatment with different concentrations of GAS before A/R and the 20 mg/L GAS + A/R group exhibited the highest cell viability. Therefore, the optimal concentration of GAS of 20 mg/L was chosen for subsequent experiments. In Figure 1(b), our data demonstrated that siRNA14-3-3*η* could inhibit this effect significantly by GAS.

### 3.2. Effect of GAS on Cardiomyocyte CPK and LDH Levels Induced by A/R

**Figure 2:**
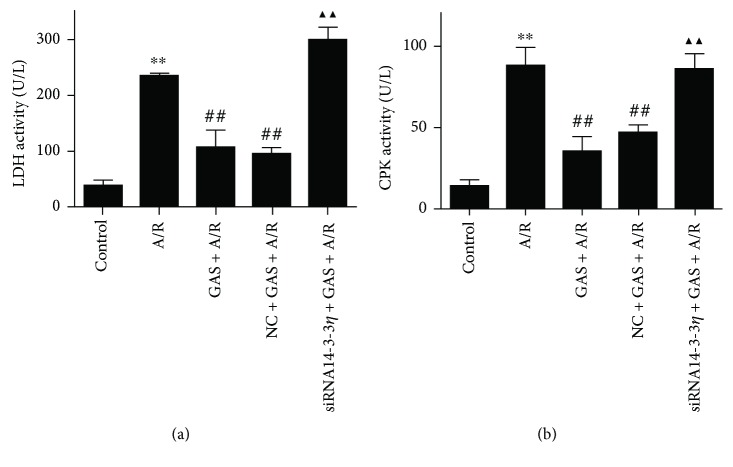
Effects of GAS on LDH and CPK activities in the H9c2 cells after A/R. Data are expressed as the mean ± SEM (*n* = 3). ^∗∗^*P* < 0.01 versus control group; ^##^*P* < 0.01 versus A/R group; ^▲▲^*P* < 0.01 versus GAS + A/R group.

After A/R treatment, the degree of damage to the cell membrane during A/R was analyzed by the activity of CPK and LDH in the culture medium. As shown in Figure 2, compared with the control group, the activities of LDH and CPK in the A/R group increased significantly. Pretreatment with GAS significantly decreased the levels of CPK and LDH activities relative to the A/R group. In addition, upon transfection with siRNA14-3-3*η*, the CPK and LDH activity levels increased significantly.

### 3.3. GAS Inhibits the Cardiomyocyte Apoptosis Induced by A/R

**Figure 3:**
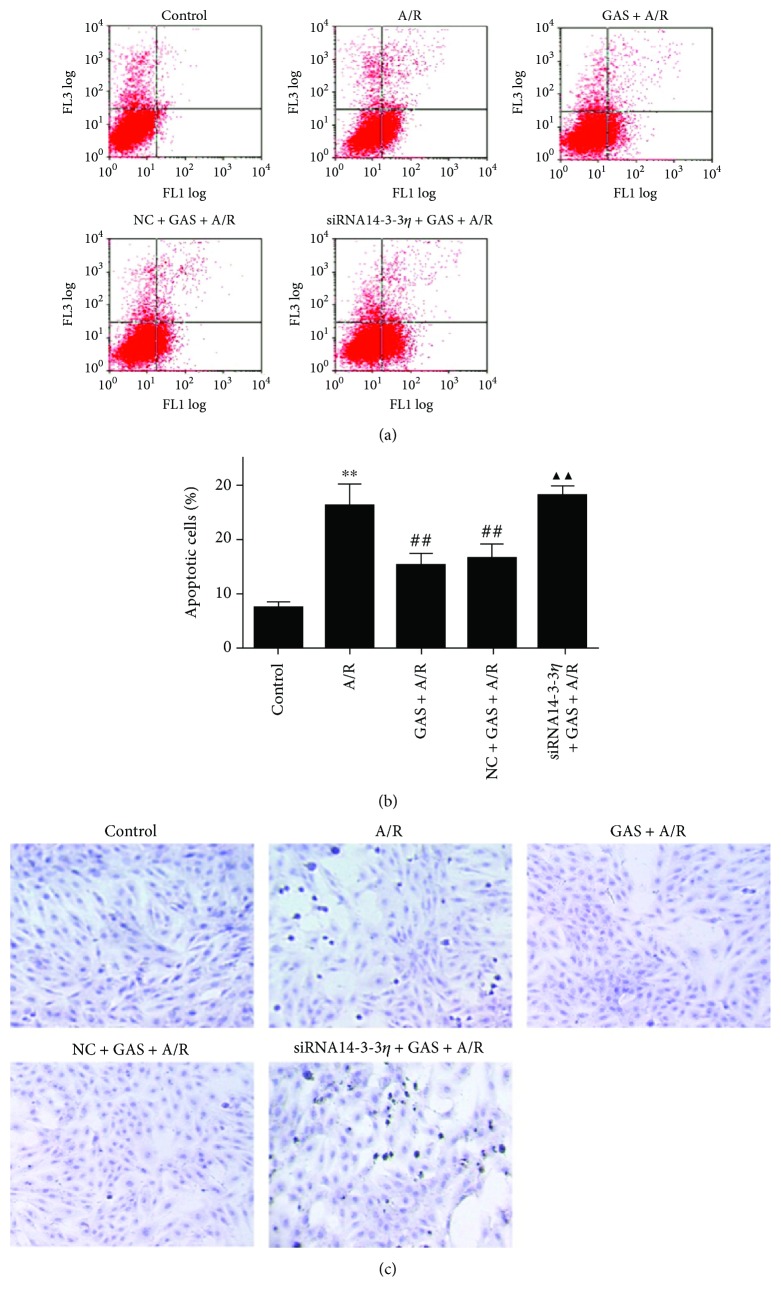
The effects of pretreatment with GAS inhibited the apoptosis of cardiomyocytes exposed to A/R. (a) Representative dot plots of PI (*y*-axis) versus annexin V (*x*-axis) by flow cytometry. (b) The quantitative analysis of apoptotic cell populations. The values are expressed as the means ± SEM (*n* = 3). ^∗∗^*P* < 0.01 versus control group, ^##^*P* < 0.01 versus A/R group, and ^▲▲^*P* < 0.01 versus GAS + A/R group. (c) Apoptotic cardiomyocytes were detected by TUNEL, and apoptotic cells are stained brown.

The apoptosis of each group was detected by flow cytometry using annexin V-FITC and PI double staining (Figures 3(a) and 3(b)), and the TUNEL assay (Figure 3(c)) further verified the apoptosis results. Increased apoptotic cells occurred with anoxia reoxygenation injury, and GAS inhibited the increase in the apoptosis, which is induced by the A/R. The antiapoptotic effect of GAS was cancelled after transfection with siRNA14-3-3*η*.

### 3.4. GAS Decreases the ROS Levels in H9c2 Cells Induced by A/R

**Figure 4:**
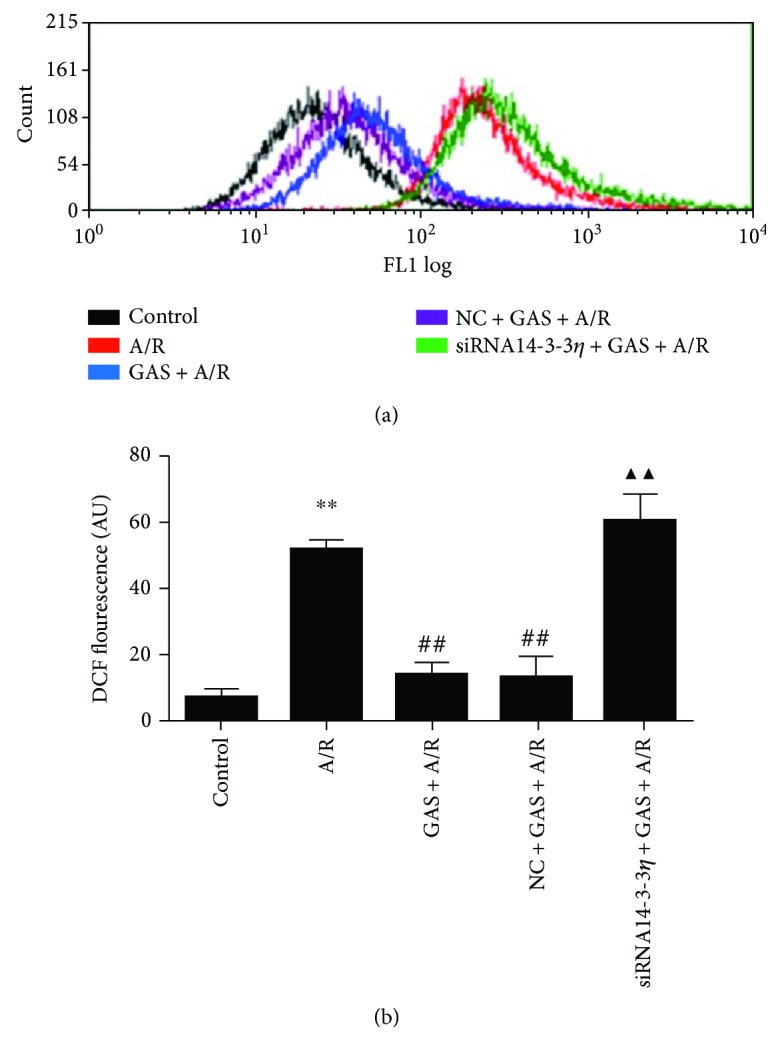
Gastrodin (GAS) pretreatment decreased the ROS generation of H9c2 cells induced by A/R, while siRNA14-3-3*η* abrogated this effect. (a) DCF fluorescence analysis by flow cytometry. (b) Column bar graph of cell fluorescence for DCF. Data are expressed as the mean ± SEM (*n* = 3). ^∗∗^*P* < 0.01 versus control group, ^##^*P* < 0.01 versus A/R group, and ^▲▲^*P* < 0.01 versus GAS + A/R group.

ROS can attack cellular components and provoke apoptosis and necrosis [17]. A DCFH-DA fluorescence probe is used to analyze the intracellular ROS level in cardiomyocytes. Our results show that ROS of the A/R group increased significantly, GAS pretreatment significantly decreased the ROS level that was induced by A/R, and this effect of GAS decreasing the active ROS in the H9c2 cells was blocked by siRNA14-3-3*η* (Figure 4).

### 3.5. Pretreatment with GAS Upregulates 14-3-3*η* Expression in Cardiomyocytes Subjected to A/R

**Figure 5:**
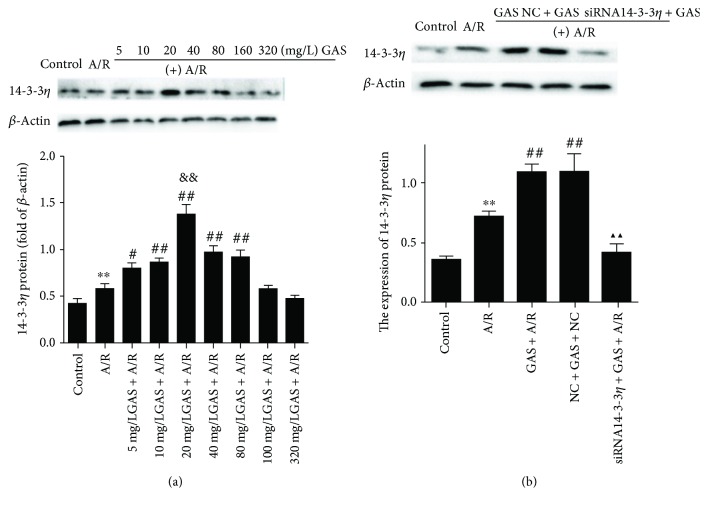
The effect of gastrodin (GAS) on the expression of 14-3-3*η* in H9c2 cells exposed to anoxia/reoxygenation (A/R) injury. 14-3-3*η* expression was evaluated by Western blot, and *β*-actin was used as an internal control. (a) The expression of the 14-3-3*η* protein significantly increased in the A/R group (^∗∗^*P* < 0.01 versus control group). In the concentration-effect GAS + A/R group, the expression of 14-3-3*η* in the GAS + A/R group was higher than that in the A/R group (^#^*P* < 0.05 and ^##^*P* < 0.01 versus A/R group), and the 20 mg/L GAS + A/R group presented with the highest expression of 14-3-3*η* (^&&^*P* < 0.01 versus 5, 10, 40, 80, 160, and 320 mg/L GAS + A/R group). (b) The expression of the 14-3-3*η* protein significantly increased in the A/R group (^∗∗^*P* < 0.01 versus control group). And the expression of the 14-3-3*η* protein significantly increased in both the GAS + A/R group and the NC + GAS + A/R group (^##^*P* < 0.01 versus A/R group) but decreased in the siRNA14-3-3*η* + GAS + A/R group (^▲▲^*P* < 0.01 versus GAS + A/R group). Data are expressed as the mean ± SEM (*n* = 3).

To investigate if 14-3-3*η* is involved in the protective effects of GAS on A/R-induced cardioprotection and to determine the optimal concentration of GAS, the protein level of 14-3-3*η* was analyzed. Before executing anoxia reoxygenation injury, H9c2 cells were pretreated with GAS at different concentrations (5, 10, 20, 40, 80, 160, and 320 mg/L) for 24 h. As shown in Figure 5(a), the expression of 14-3-3*η* with 20 mg/L GAS was shown to have the highest expression of 14-3-3*η*. The optimal concentration and precondition time are similar to the results of MTS. In addition, the level of 14-3-3*η* pretreated with GAS increased significantly; however, when transfected with siRNA14-3-3*η*, these protective effects were abolished (Figure 5(b)).

### 3.6. Measurement of the Mitochondrial Membrane Potential (∆*Ψ*m)

**Figure 6:**
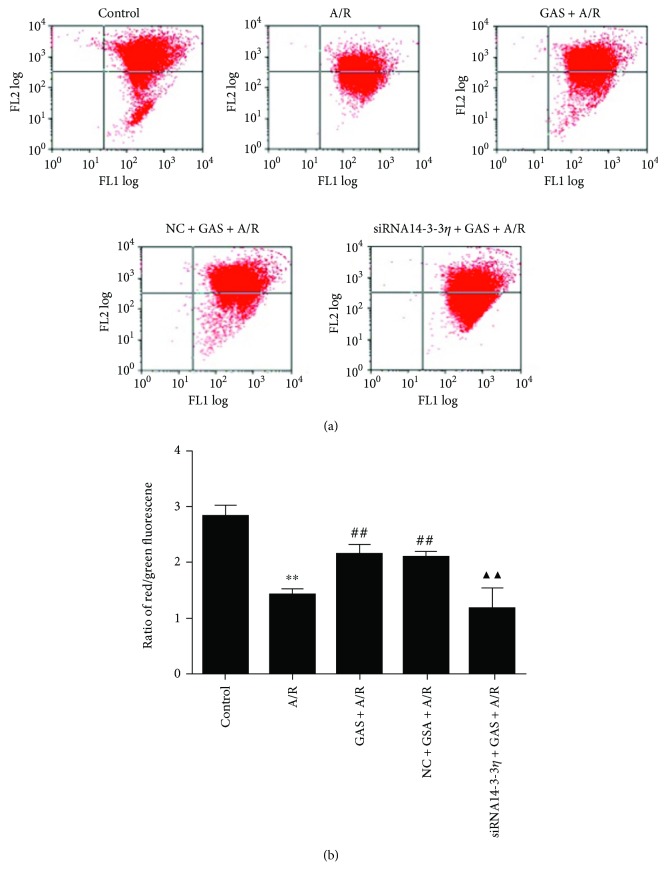
Effects of GAS pretreatment on the loss of the mitochondrial membrane potential in cardiomyocytes induced by A/R. (a) Representative point diagram of flow cytometry. (b) ∆*Ψ*m was calculated by the proportion of red/green fluorescence acquired from flow cytometry. Data are expressed as the mean ± SEM (*n* = 3). ^∗∗^*P* < 0.01 versus control group; ^##^*P* < 0.01 versus A/R group; ^▲▲^*P* < 0.01 versus GAS + A/R group.

The maintenance of the mitochondrial membrane potential (∆*Ψ*m) is greatly significant for mitochondrial integrity and bioenergetic function [18]. JC-1 is the only fluorescent cationic dye that causes aggregates and forms a red fluorescence peak at a high mitochondrial membrane potential, whereas JC-1 exists as a monomer form and emits a green fluorescence peak at a low mitochondrial membrane potential. The change from red to green fluorescence indicates a loss of the mitochondrial membrane potential.  Figure 6 shows progressive loss of red JC-1 aggregate fluorescence of the A/R-treated cells, and GAS pretreatment can significantly alleviate the loss of red fluorescence. However, the effect of GAS was reversed by transfection with siRNA14-3-3*η*.

### 3.7. Effects of GAS on the Activity of Caspase-3 Induced by A/R

**Figure 7:**
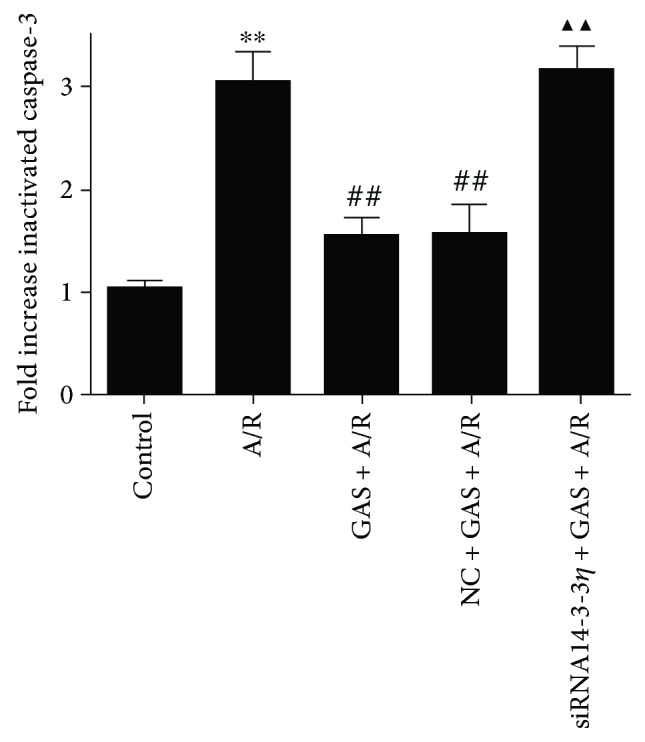
Gastrodin (GAS) pretreatment influenced the activity of caspase-3 induced by A/R injury. The column bar graph shows the activity of caspase-3 in different groups. Data are shown as the mean ± SEM (*n* = 3). ^∗∗^*P* < 0.01 versus control group, ^##^*P* < 0.01 versus A/R group, and ^▲▲^*P* < 0.01 versus GAS + A/R group.

To test the effects of GAS pretreatment on the A/R-induced changes in the activity of caspase-3, cytoplasmic proteins were collected to evaluate the activity of caspase-3. Programmed cell death can be initiated by several factors, which then converge to a common and combined biochemical caspase pathway that critically involves the activation of caspase-3 and results in the execution of apoptosis [19, 20]. As shown in Figure 7, compared with the control group, the activity of caspase-3 was significantly increased in the A/R group, and the increased activity of caspase-3 was significantly inhibited by GAS. However, this effect was abolished by transfecting H9c2 cells with the siRNA14-3-3*η* adenovirus.

### 3.8. GAS Pretreatment Inhibits the mPTP Induced by A/R in Cardiomyocytes

**Figure 8:**
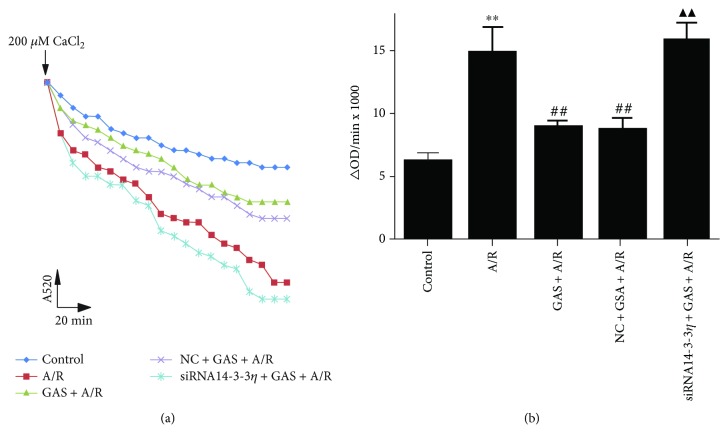
The effects of gastrodin (GAS) preconditioning inhibited mPTP opening in H9c2 cells induced by A/R. (a) The opening of mPTP was reflected by absorbance at a wavelength of 520 nm after adding 200 mM CaCl_2_ and monitoring over 20 minutes. (b) Changes in the absorbance value at 520 nm/min (∆OD·min^−1^) were used to express the level of mPTP opening (∆OD = A520_0 min_ − A520_20 min_). Data are expressed as the mean ± SEM (*n* = 3). ^∗∗^*P* < 0.01 versus control group, ^##^*P* < 0.01 versus A/R group, and ^▲▲^*P* < 0.01 versus GAS + A/R group.

Mitochondria play an important role in cell life by regulating both survival and death signaling pathways. Mitochondria are the main regulators of cell death through apoptosis, necrosis, and autophagy. We detected the absorbance to analyze the extent of mPTP opening at a 520 nm/min (OD·min^−1^) rate (Figure 8(a)). The results indicate that A/R significantly increased the opening of mPTP, but this change was significantly inhibited by GAS pretreatment, and the effects of GAS were limited by siRNA14-3-3*η* (Figure 8(b)).

## 4. Discussion

Gastrodin is a major active ingredient of *Gastrodia elata* Blume, which has been reported to have anti-inflammatory and antiapoptotic roles in various diseases [21, 22]. Oxidative stress plays an important role in the brain damage induced by ischemia and reperfusion. Inflammation and oxidative stress are tightly intertwined. Inflammation is a cellular response to factors (including those due to oxidative stress) that challenge the homeostasis of the tissues, but this process also acts as a defense mechanism to maintain the equilibrium of the functions [23]. Many reports show that gastrodin can play an important role in multiple pathways, including ERK1/2, p38MAPK, GATA-4, NF-*κ*B, iNOS, COX-2, and cytokines [24–26]. However, the effects of gastrodin in cardiac A/R-induced injury and the related signaling mechanisms remain unclear. In this study, we found that GAS preconditioning significantly increased cell viability and decreased the LDH and CPK released in the H9c2 cells. The highest cell viability was 20 mg/L, suggesting that GAS protects the myocardium against A/R-induced injury, and the optimum concentration and the preconditioning time are 20 mg/L and 24 h, respectively. In addition, all of these effects of GAS are antagonized by transfection with siRNA14-3-3*η*.

In the present study, we confirm that GAS could significantly increase the expression of the 14-3-3*η* protein in H9c2 cells suffering from A/R. 14-3-3*η* is a member of the 14-3-3 family. 14-3-3 proteins are intracellular, dimeric, phosphoserine-binding molecules that play a significant role in signal transduction, apoptosis, and checkpoint control pathways through binding to many signaling proteins such as Raf1, protein kinase C, Ask-1, cdc25c, BAD, and forkhead transcription factor1 (FKHRL1). Via enzymatic binding with Ask-1, a MAPKKK, 14-3-3 stops the activation of JNK and p38 MAPK. Therefore, most of the studies on 14-3-3 proteins demonstrate their protective function against apoptotic cell death. It has been reported that the 14-3-3*η* protein in the heart is a potential target through which we can reduce the pathological outcomes of MI [27]. Our results show that the expression of the 14-3-3*η* protein is highest in the 20 mg/L group. To investigate if the cardioprotective effects of GAS are related to 14-3-3*η* in A/R-induced injury, transfection with siRNA14-3-3*η* was performed to investigate if these effects could be eliminated with downregulation of 14-3-3*η*. These protective effects are abolished when cells are transfected with siRNA14-3-3*η*, which suggests that the cardioprotective effects of the GAS are related to the 14-3-3*η* protein.

A/R, as an I/R experimental model, is widely used in laboratories. According to previous studies, A/R has been confirmed to be able to cause obvious apoptosis in cultured cardiomyocytes, which can be detected by flow cytometry. A/R-induced injury is caused by oxidative stress, and oxidative stress-mediated ROS are tightly linked with cardiac dysfunction [28, 29]. The imbalance between the production and purging of ROS can result in irreversible damage to cells, finally leading to cell apoptosis [30]. It is known that GAS ameliorates cerebral damage after transient focal cerebral ischemia by promoting the ability to reduce ROS damage in vivo and hippocampal neuronal death and excitotoxicity in vitro [31, 32]. Cardiac ischemia-reperfusion generates excessive ROS, which is an important mechanism following cardiomyocyte apoptosis. Our study shows that GAS can significantly reduce ROS generation, and this effect is abolished once cardiomyocytes are transfected with siRNA14-3-3*η*.

Impairment of mitochondria is a threat to proper cellular function, because it leads to a lack of energy generation and the redundant release of ROS [33].The direct relationship between mitochondrial Δ*Ψ* and ROS production [34, 35] ensures the foundation for the notion that uncoupling of oxidative phosphorylation abates mitochondrial O_2_^−^ production [36, 37]. Previous studies have found that a change in the mitochondrial membrane potential is a significant index of mitochondrial dysfunction [38]. In the current study, we separate the cytoplasm and mitochondrial components of cardiomyocytes to evaluate the degree of mPTP opening and use JC-1 to assess the mitochondrial membrane potential. We found that A/R-induced injury not only affected the opening of the mPTP but also led to disruption in the mitochondrial membrane potential, matrix swelling, and outer membrane rupture. We have confirmed that GAS can inhibit mPTP opening, decreasing disruption of the mitochondrial membrane potential and ultimately decreasing H9c2 cell apoptosis. And when transfected with siRNA14-3-3*η*, the protective effects of GAS on H9c2 cells are eliminated. All of these findings suggest that the cardioprotective effect of GAS in H9c2 cells subjected to A/R is related to the mitochondrial pathway.

Mitochondrial dysfunction causes the apoptotic signal cascade, and engaged cellular targets lead to apoptosis [39]. Caspase-3, one of the key effectors in cell apoptosis, is initiated and plays an important role in the mitochondria-mediatedapoptosis [40]. In our study, GAS preconditioning significantly reduced caspase-3 activity caused by A/R injury, ultimately decreasing cardiomyocyte apoptosis, and these effects of GAS are antagonized by transfection with siRNA14-3-3*η*.

The current study has proved that GAS has protective effects on A/R-induced injury in H9c2 cells. GAS can reduce the generation of ROS, alter the preservation of the mitochondrial membrane potential, inhibit mPTP opening, decrease the activation of caspase-3, and ultimately decrease cardiomyocyte apoptosis; all of these effects were abolished by siRNA14-3-3*η*. These findings suggest that GAS protects cardiomyocytes against A/R injury via the 14-3-3*η* protein.

## Data Availability

Interested readers can reproduce our results by using our algorithm.
